# Renal Artery Stenosis—When To Screen, What To Stent?

**DOI:** 10.1007/s11883-014-0416-2

**Published:** 2014-04-18

**Authors:** Claudine G. Jennings, John G. Houston, Alison Severn, Samira Bell, Isla S. Mackenzie, Thomas M. MacDonald

**Affiliations:** 1 Medicines Monitoring Unit and Hypertension Research Centre, Ninewells Hospital and University of Dundee, Dundee, DD1 9SY UK; 2 Department of Renal Medicine, Ninewells Hospital, Dundee, DD1 9SY UK; 3 Department of Radiology, Ninewells Hospital, Dundee, DD1 9SY UK

**Keywords:** Renal artery stenosis, Atherosclerosis, Fibromuscular dysplasia, Percutaneous transluminal renal artery angioplasty

## Abstract

Renal artery stensosis (RAS) continues to be a problem for clinicians, with no clear consensus on how to investigate and assess the clinical significance of stenotic lesions and manage the findings. RAS caused by fibromuscular dysplasia is probably commoner than previously appreciated, should be actively looked for in younger hypertensive patients and can be managed successfully with angioplasty. Atheromatous RAS is associated with increased incidence of cardiovascular events and increased cardiovascular mortality, and is likely to be seen with increasing frequency. Evidence from large clinical trials has led clinicians away from recommending interventional revascularisation towards aggressive medical management. There is now interest in looking more closely at patient selection for intervention, with focus on intervening only in patients with the highest-risk presentations such as flash pulmonary oedema, rapidly declining renal function and severe resistant hypertension. The potential benefits in terms of improving hard cardiovascular outcomes may outweigh the risks of intervention in this group, and further research is needed.

## Introduction

Renal artery stenosis (RAS) is defined as narrowing of main or branch renal arteries and is most often caused by atherosclerotic disease or fibromuscular dysplasia (FMD). Notable rarer causes include large vessel vasculitis (typically Takayasu arteritis), renal artery aneurysm, extrinsic compression and congenital malformations [1]. RAS may lead to the development of renovascular disease and significant associated morbidity in terms of hypertension and nephropathy. Atherosclerotic RAS is also associated with adverse cardiovascular outcomes. Despite extensive research in this area, the investigation, diagnosis and management of RAS remain controversial. The clinical significance of RAS differs between patients, and the indications for revascularisation are increasingly limited when outcomes are compared with those of modern medical management. This review will focus on the two main causes of RAS, namely atherosclerosis and FMD, and will summarise the latest evidence in this area, including the much anticipated results of the Cardiovascular Outcomes in Renal Atherosclerotic Lesions (CORAL) study [2].

## Atherosclerotic RAS

Atherosclerotic RAS typically affects the most proximal portion of the renal artery, with build-up of atherosclerotic plaque leading to narrowing of the arterial lumen. The reported prevalence of atherosclerotic RAS differs widely, and atherosclerotic RAS is often found in high-risk patients with other evidence of atheromatous disease. Approximately 20 % of patients have bilateral disease or disease affecting a single functioning kidney, but most patients have unilateral disease [2, 3]. A general population of 870 participants in the Cardiovascular Health Study were screened using duplex ultrasonography, and the prevalence of RAS was 6.8 % with a mean age of 78.7 years [4]. In high-risk populations the prevalence can be significantly higher, with data from some centres showing 18 % of patients undergoing coronary angiography and 45 % of patients undergoing peripheral angiography have evidence of RAS [5, 6].

## Fibromuscular Dysplasia

FMD is recognised as the commonest cause of RAS after atherosclerosis, and is thought to account for between 5 and 10 % of patients with renovascular disease. FMD is a non-atherosclerotic, non-inflammatory vascular disease that can affect any arterial bed, although it most commonly affects the renal and carotid arteries [7]. FMD typically involves medial and distal portions of the renal artery [8]. Classification is based on findings on angiography and broadly divides FMD into multifocal (mostly medial disease with the classic “string of beads” appearance) and unifocal (single stenosis shorter than 1 cm) or tubular (stenosis at least 1 cm in length) subtypes [9]. Angiographic classification is important, as a recent systematic review found that patients with non-medial disease had better blood pressure outcomes than patients with medial disease after angioplasty [10]. The cause of FMD is unknown, although around 10 % of cases are thought to be familial [11, 12]. Smoking is closely associated with FMD, but direct causality has not been proven [13].

The prevalence of renal FMD is estimated at four per 1,000 population, although the true prevalence may be considerably higher [7]. Screening investigations in potential renal donors have found an FMD prevalence of 3.8 %, and a retrospective cohort study of 2,640 living renal donors (of which 87 % were female with a mean age of 52 years) showed a prevalence of 2.6 % [14–16]. Only around one third of patients with incidental FMD in these cohorts had a history of hypertension. A US registry of FMD was commenced in 2008 and currently has 447 recorded patients, of whom 294 have renal involvement (the rest having predominantly carotid disease); the mean age at diagnosis was 51.9 years, 91 % were female and 72 % had a history of hypertension [12].

## The Renovascular Syndrome

The potential clinical consequences of RAS include renovascular hypertension and ischaemic nephropathy. The reduction in renal perfusion attributable to RAS triggers a series of hormonal and neuronal responses that raise systemic blood pressure and therefore compensate for reduced renal blood flow. This is seen most notably in activation of the renin–angiotensin–aldosterone system (RAAS). Haemodynamic effects of stenotic lesions generally only become significant when severe stenosis overcomes these compensatory mechanisms, and this generally requires at least 80 % reduction in the luminal diameter of the artery [17, 18].

Renovascular hypertension is the commonest secondary cause of hypertension; however, essential hypertension still accounts for approximately 95 % of hypertensive patients and RAS will often be an incidental finding, particularly in older patients. RAS is also often cited as a cause of renal function decline and potentially end-stage renal disease. The UK Renal Registry reports that 6.9 % of incident dialysis was due to renovascular disease, and a 20-year follow-up of patients starting dialysis in a single French centre found that 12 % had documented evidence of RAS [19, 20]. It is not clear how renovascular disease was diagnosed, and patients with a history of hypertension and declining renal function are often labelled as having renovascular disease with limited or no evidence of direct causality. Identification of an asymmetrically small kidney in patients older than 50 years has a 70 % chance of being due to significant atherosclerotic stenosis (but it may be entirely asymptomatic) [11]. Flash pulmonary oedema is also classically associated with bilateral RAS where there is hypertension and extracellular fluid volume excess due to impaired pressure natriuresis [21, 22]. RAS should be suspected in patients with recurrent episodes of pulmonary oedema, particularly if there is preserved left ventricular function on echocardiography.

The conditions that make up the renovascular syndrome may be found alone or in combination, and all are multifactorial; therefore, establishing a direct causal relationship with RAS is often difficult. In an individual patient, the risk of adverse outcomes due to RAS is dependent on the degree of stenosis, the rate of progression and associated comorbidities, none of which are easily defined or predictable. Significantly increased cardiovascular mortality is associated with atherosclerotic RAS, and in one follow-up study of patients undergoing coronary angiography an incidental finding of renal artery lesions was an independent risk factor for mortality, which approached 30 % at 4 years [23]. This is presumably due to the high burden of atherosclerotic disease in these patients.

Determining the likely progression of atherosclerotic lesions causing RAS is important when making management decisions as progression to occlusion is not inevitable. A prospective study of 295 kidneys in 170 patients found that the cumulative incidence of progression was 51 % at 5 years, and only nine patients underwent progression to complete occlusion (significant risk factors for progression were greater baseline stenosis, systolic hypertension and history of diabetes) [24]. Another study found that over a 5-year follow-up period, progressive narrowing of the stenotic diameter was approximately 5 % per year and only around 50 % of arteries showed progression [25]. Progression was not linear, but occurred erratically and often rapidly, likely due to plaque rupture or subintimal bleeding. There are very few data on the progression of lesions in FMD; however, the risk of hypertension increases with age, and there is some evidence that the disease follows a more aggressive course in smokers [7, 10, 13].

## Which Patients Should Be Investigated for RAS?

Guidelines regarding who to investigate for atherosclerotic RAS were published by the American College of Cardiology/American Heart Association in 2005 and recommend screening virtually all hypertensive patients, patients with declining renal function, patients with discrepancy in renal size and patients with sudden, unexplained pulmonary oedema [22]. These guidelines predate a number of key clinical trials which have substantially changed how atherosclerotic RAS is managed. Current clinical consensus would generally not advocate screening for atherosclerotic RAS in everyone, particularly where it will not the alter management plan. Suggested criteria for which patients to investigate are outlined below:

1. Hypertension
  Rapid onset of hypertension over the age of 55 years with additional adverse features (difficult-to-control blood pressure, association with decline in renal function, association with heart failure)
  Malignant, accelerated or drug-resistant hypertension
2. Deteriorating renal function
  Rapid, unexplained decline in renal function (more than 50 % increase in serum creatinine concentration over 12 months)
  Significant deterioration in renal function after introduction of an ACE inhibitor or angiotensin II receptor blocker
3. Repeated hospital admissions for heart failure with preserved left ventricular function on echocardiography

FMD expert consensus guidelines suggest that screening for FMD should be undertaken in the investigation of hypertension diagnosed before the age of 30 years, where there is malignant (grade 3) hypertension, refractory hypertension (hypertension despite triple-drug therapy including a diuretic) or hypertension associated with a small kidney [22]. In view of the mean age of patients diagnosed with FMD being over 50 years, it is also recommended that screening could be considered in older hypertensive patients [7].

## Investigation of RAS

The choice of investigation will depend on the clinical situation and local expertise. The gold standard investigation remains catheter digital subtraction angiography as it provides accurate anatomical information, an assessment of renal perfusion and the option of measuring the pressure gradient across the stenotic region to provide information on the functional significance of a demonstrated stenosis. With increasing availability of high-specification imaging techniques, most centres now use non-invasive imaging as the first-line investigation. Duplex ultrasonography is cheap and safe and is particularly useful as a screening tool to exclude FMD in patients with low probability of disease and in patients where other forms of imaging are contraindicated (because of concerns such as with contrast medium administration in patients with renal impairment). A meta-analysis showed duplex ultrasonography has 85 % sensitivity and 82 % specificity for detection of RAS [26], and estimation of the functional severity of the stenosis is possible by Doppler measurement of renal artery velocity. Use may be limited in obese patients, and there is potential for images to be obscured by bowel gas. The results are also highly dependent on operator skill [7, 26, 27]. Comparisons of catheter angiography and duplex ultrasonography have found that ultrasound scans will generally overestimate the degree of stenosis present [28]. Both contrast-enhanced CT and MRI provide good anatomical information, and the diagnostic sensitivity and specificity have been shown to be 94 % and 93 % for CT angiography and 90 % and 94 % for magnetic resonance angiography [29–32]. A meta-analysis showed CT angiography and gadolinium-enhanced magnetic resonance angiography gave more accurate diagnoses than ultrasonography or captopril scintigraphy [33]. CT angiography has higher spatial resolution than magnetic resonance angiography and is therefore probably a better tool for investigation of FMD [7]. For both CT angiography and magnetic resonance angiography there are concerns regarding use of contrast-enhanced studies, with potential for contrast-medium-induced nephropathy after use of non-ionic iodinated CT contrast media and nephrogenic systemic fibrosis with some magnetic resonance gadolinium chelate contrast media. Both are more likely in patients with known renal impairment. As well as imaging the renal arteries for occlusive disease, MRI may be used to quantitatively assess renal perfusion using contrast-enhanced scans [34] or arterial spin labeling [35, 36]. In addition, single-kidney renal volumes may be derived from contrast-enhanced MRI which correlate well with the single-kidney glomerular filtration rate [37, 38]. Non-invasive imaging techniques have largely superseded functional renal investigations or indirect tests for RAS such as captopril renography, nuclear medicine renograms or measurements of renal vein renin.

An emerging area of interest in the investigation of RAS is use of blood-oxygen-level-dependent MRI. This non-invasive technique does not require the use of a contrast medium and is well validated as the basis for functional brain imaging techniques. It is a method of estimating oxygenation of renal tissue. A small pilot study of 23 kidneys showed 67 % sensitivity and 86 % specificity for detecting kidneys that were likely to recover function after revascularisation [39]. This study hypothesised that the ratio used in the calculations reflected metabolically active hypoxic renal tissue that was potentially salvageable following restoration of renal blood supply. Although pilot studies have shown some promising results, there are still inconsistencies in the data produced, and it is felt that more experience of how to interpret the data from these scans is required before they could be put into routine clinical use [40].

## Management Overview

The management goals in RAS are to control hypertension, preserve renal function and prevent recurrent flash pulmonary oedema. Management of FMD is reasonably clear-cut; however, management of atherosclerotic RAS continues to be debated, and it is apparent that there is no single strategy suitable for all patients. The results of a number of key clinical trials on the use of percutaneous transluminal renal artery angioplasty (PTRAA) and significant improvements in medical therapy have shifted the emphasis of management towards conservative or medical care, and the role of revascularisation is yet to be redefined.

## Management of FMD

An expert consensus statement on management of FMD states that revascularisation should be attempted only in those with evidence of symptomatic disease (renovascular hypertension or renal atrophy) [7]. One of the earliest renal angioplasty studies, in 193 patients with FMD, showed a 50 % cure rate for hypertension and the most recent cohort study showed a mean blood pressure reduction of 17/7 mmHg after 12 months’ follow-up [41, 42]. A meta-analysis of 47 angioplasty studies published in 2010 showed moderate benefit of angioplasty in terms of hypertension, with a 36 % cure rate (defined as blood pressure below 140/90 mmHg without medical therapy) [10]. Favourable outcome after angioplasty is predicted by younger age (less than 40 years) at diagnosis, hypertension of less than 5 years’ duration and systolic blood pressure below 160 mmHg [43]. There is no evidence that angioplasty for FMD improves renal function. Long-term follow-up is important for patients with an incidental finding of FMD as they are significantly more likely to develop hypertension over time (in one retrospective cohort, 26.6 % of patients with FMD developed hypertension over a 7.5-year follow-up period compared with 6.1 % of age-matched controls) [15]. Therefore, although intervention may not be appropriate at the first review, it may become appropriate over time, and delay must be balanced against older patients generally responding less well to intervention.

## Medical Management—Atherosclerotic RAS

Current best medical management involves good blood pressure control using antihypertensive medications, a statin, an antiplatelet agent and lifestyle advice, particularly regarding smoking cessation. Supporting evidence for blood pressure control in the RAS patient group is extrapolated from general populations with cardiovascular disease, chronic renal disease and diabetes. RAAS antagonists (ACE inhibitors or angiotensin II receptor blockers), calcium channel blockers and beta blockers all have supporting evidence and general acceptance for hypertension management in RAS. Historically, use of RAAS antagonists was contraindicated in this patient group owing to concerns of precipitating decline in renal function. RAAS antagonists have been used as a screening tool as patients who show a significant rise in creatinine concentration after their introduction are likely to have evidence of RAS (in a high-risk population, introduction of an ACE inhibitor was 100 % sensitive and 70 % specific for detection of severe bilateral RAS [44]). Concerns with RAAS antagonists in this patient group are probably overstated, and observational studies have shown improved outcomes using this class of antihypertensive [11, 22]. In a prospective study of 378 patients with renovascular disease, 92 % tolerated introduction of RAAS antagonists and showed reduced mortality [45]. A follow-up study of 195 patients with angiographic evidence of RAS found that those treated with RAAS antagonists had improved survival whether or not a revascularisation procedure was performed [46]. A further observational study of 3,570 Canadian patients with renovascular disease found 53 % were taking RAAS antagonists and these patients had significantly lower risk of death, myocardial infarction or stroke [hazard ratio (HR) 0.70; 95 % confidence interval (CI) 0.53–0.90] [47]. The concern with observational data is that they reflect selection bias, and those able to tolerate RAAS antagonists have less significant disease and would have better outcomes anyway. Despite the lack of randomised trials, there is a growing consensus that RAAS antagonists should be used in patients with RAS but should be introduced cautiously with careful monitoring.

Statin therapy has become a mainstay of treatment for all atherosclerotic vascular disease [22]. Experimental models in rats with RAS have shown that simvastatin reduces renal fibrosis by upregulating antifibrogenic mediators and has beneficial effects through antioxidant, anti-inflammatory and antifibrotic actions [48]. These actions are seen in addition to lowering of lipid concentrations, and may explain why statins are so effective in treating cardiovascular disease. Observational data supporting statin therapy in RAS patients come from a follow-up review of the data from the Single Operator, Single Center, Renal Stent Retrospective Study (SOCRATES) of 748 patients who underwent renal artery stenting which found decreased mortality for those prescribed a statin (HR 0.69; 95 % CI 0.47–0.98) [49]. A retrospective study of 104 patients with atherosclerotic renal disease showed that over an 11-year follow-up period those receiving statin therapy had significant reduction in renal end points (7.4 % vs 38.9 %) and overall mortality (5.9 % vs 36.1 %) [50]. Cheung et al. [51] showed that in 79 patients who had serial renal angiograms, the risk of progression of RAS over a mean 28-month follow-up period was reduced by statin therapy (relative risk 0.28; 95 % CI 0.10–0.77) and they postulated that statin therapy could alter the natural history of atherosclerotic renovascular disease. These results indicate that statins reduce mortality and limit lesion progression and should be considered in all patients with confirmed or suspected atherosclerotic RAS.

Use of antiplatelet agents and smoking cessation in patients with atherosclerotic RAS has no specific supporting evidence, but observational data looking at outcomes from other forms of vascular disease, particularly peripheral vascular disease and coronary disease, suggest that both strategies have merit, and these results could be extrapolated to the RAS population [52–56].

## Atherosclerotic RAS Management—Revascularisation

Surgical revascularisation has largely been replaced by PTRAA after trials in the early 1990s showed improved safety with equal efficacy for balloon angioplasty compared with surgical intervention [57]. Subsequent comparisons of angioplasty with and without stenting for atheromatous disease showed increased long-term renal artery patency with use of stents, and therefore stenting became a first-line treatment [58–60]. Revascularisation of renal arteries with PTRAA became extremely popular, and between 1996 and 2000 the number of PTRAAs performed for RAS quadrupled in the USA [61, 62]. The aim of revascularisation is to reduce obstruction to renal perfusion, thereby improving downstream ischaemia and reducing activation of RAAS, resulting in improved blood pressure control and preservation of renal function. The difficulty lies in knowing which patients will benefit from revascularisation as a high degree of stenosis may not correlate with significant haemodynamic effects and RAS may be an incidental finding [63]. A number of randomised clinical trials have now looked at outcomes for blood pressure, renal function and cardiovascular mortality, comparing revascularisation and best medical therapy against best medical therapy alone. A summary of the most significant trials is presented in Table 1, and the two largest trials, the Angioplasty and Stenting for Renal Artery Lesions (ASTRAL) trial [3] and CORAL trial [2] trial, are discussed further below.

**Table 1:** Summary of randomised controlled trials for revascularisation versus medical therapy in the management of atherosclerotic renal artery stenosis (*RAS*)

Study	Population	Intervention	Findings	Reference
Scottish and Newcastle Renal Artery Stenosis Collaborative Group (1998)	135 participants, >40 years, hypertension, RAS >50 %	PTRAA and medical therapy vs medical therapy alone	Significant fall in BP after PTRAA in bilateral RAS only	[64]
Dutch Renal Artery Stenosis Intervention Study Group (2000)	106 participants, hypertension, RAS >50 %	PTRAA and medical therapy vs medical therapy alone	No difference in BP or renal function outcomes between groups	[65]
STAR (2009)	140 participants, CrCl <80 ml/min, RAS >50 %	PTRAA with stenting and medical therapy vs medical therapy alone	Stent placement had no impact on renal function. Significant complications with procedures	[66]
ASTRAL (2009)	806 participants, RAS >60 %, uncertainty as to benefit of revascularisation	PTRAA with or without stenting and medical therapy vs medical therapy alone	No difference in BP, renal function or mortality between groups	[3]
CORAL (2013)	947 participants, hypertension and CKD, RAS >80 % (or 60–80 % with pressure gradient)	PTRAA with stenting and medical therapy vs medical therapy alone	No difference in incidence of CV and renal events or all-cause mortality. 2-mmHg improvement in systolic BP in stent group	[2]

*ASTRAL* Angioplasty and Stenting for Renal Artery Lesions, *BP* blood pressure, *CKD* chronic kidney disease, *CORAL* Cardiovascular Outcomes in Renal Atherosclerotic Lesions, *CrCl* creatinine clearance, *CV* cardiovascular, *PTRAA* percutaneous transluminal renal artery angioplasty, *STAR* Stent Placement and Blood Pressure and Lipid-Lowering for the Prevention of Progression of Renal Dysfunction Caused by Atherosclerotic Ostial Stenosis of the Renal Artery

Intervention with revascularisation is also not without risk, and complications after PTRAA include haematoma at the puncture site, renal injury due to contrast media, cholesterol emboli and rarely renal artery dissection. One large case series reported 30-day mortality after PTRAA of 2.2 % (all deaths occurred in patients with atheromatous disease) [67]. In the Stent Placement and Blood Pressure and Lipid-Lowering for the Prevention of Progression of Renal Dysfunction Caused by Atherosclerotic Ostial Stenosis of the Renal Artery (STAR) trial, there were two procedure-related deaths among 46 patients undergoing renal artery stenting [66].

Publication of the results from the ASTRAL trial [3] in 2009 played a pivotal role in discouraging clinicians from recommending revascularisation, and the message from ASTRAL trial has now been consolidated with the results from the CORAL trial [2] published in 2013. ASTRAL randomised 806 patients with evidence of renovascular disease and substantial anatomical atherosclerotic stenosis in at least one renal artery. An additional inclusion criterion was that the patient’s physician felt uncertain about the benefit of revascularisation. Patients were randomised to receive either medical treatment with a statin, an antiplatelet agent and optimal blood pressure control or medical treatment with revascularisation (either angioplasty alone or angioplasty and stenting without the use of a renal protection device). After 5 years’ follow-up, there were no significant improvements in blood pressure or reductions in the incidence of renal or cardiovascular events or mortality in the revascularisation group, and the benefits in terms of renal function were not clinically significant. There was also a procedure-related serious complication rate of 5 %. The investigators concluded that there was some risk of harm and revascularisation did not provide any benefit above that of medical therapy [3].

CORAL [68] randomised 947 patients with atherosclerotic RAS and either hypertension or chronic kidney disease to receive optimal medical therapy [angiotensin II receptor blocker (with or without thiazide with or without amlodipine), an antiplatelet agent and atorvastatin] or optimal medical therapy with revascularisation and stent placement (with use of an embolic protection device at the operator’s discretion). CORAL required demonstration of stenosis of more than 80 % by conventional CT or magnetic resonance angiography or Doppler ultrasonography, or stenosis of 60-80 % with evidence of a 20 % pressure gradient across the stenotic lesion on angiography. The CORAL inclusion criteria should have selected patients with clinically significant stenosis; however, mean stenosis in these patients was 68 % and only 39 % of patients had stenosis of more than 80 % (which may have been overestimated depending on the imaging technique used). After a median 43-month follow-up period there was no difference between the groups for the primary composite end point (cardiovascular and renal adverse events), for the individual components of the end point or for all-cause mortality. The degree of stenosis at screening did not influence the outcomes. There was a statistically significant difference of around 2 mmHg in favour of the stent group for systolic blood pressure control. Eleven patients (2.4 %) in the stent group had a serious complication in the form of renal artery dissection. The CORAL investigators similarly concluded that there was no benefit to patients from revascularisation and there was some risk of harm.

The use of revascularisation in management of flash pulmonary oedema is supported by some evidence from older case series [69, 70]. In one series of patients with proven RAS, 41 % of patients with bilateral disease had a history of pulmonary oedema, and following stenting, 77 % of these patients had no further episodes of pulmonary oedema [71]. A recent systematic review included data from 173 patients and concluded that the quality of evidence was low and justified only a weak recommendation for PTRAA with or without stenting in patients with atherosclerotic RAS and either flash pulmonary oedema or chronic heart failure and renal insufficiency [72].

The interpretation of the results of the large trials so far in the management of atherosclerotic RAS has been limited by patient selection and methodological issues including crossover from medical to intervention arms and analysis based on intention to treat (when not all randomised patients actually underwent a procedure). ASTRAL was criticised for including patients who would never normally have been considered for revascularisation, i.e. patients for whom there was insufficient clinical evidence that the degree of stenosis identified was causing significant adverse outcomes and who, therefore, would never be expected to benefit from such a procedure. CORAL, although more robust in its patient selection, still included patients with stable disease and has similarly been criticised as proving only that treating non-significant lesions does not significantly change outcomes.

## The Future of Revascularisation in RAS

The concern now is that the entire idea of revascularisation will be discarded when actually what is needed is a new perspective on patient selection. Case reports and case series have shown that there is a group of patients who appear to benefit from revascularisation; however, at present we have no method of reliably preselecting these patients. Radiological estimations of the degree of stenosis are unreliable and correlate poorly with outcomes, rate of progression of stenosis is unpredictable and biomarkers such as renin and B-type natriuretic peptide are not specific or sensitive enough [73–75]. Future decisions on revascularisation are likely to be made on an individual patient basis, with clinical presentation as the key determinant for the management strategy adopted.

Revascularisation is not a risk-free procedure; therefore, it is likely that only patients perceived to be at high risk of an adverse outcome owing to RAS will be considered for intervention. A retrospective analysis of a patient cohort from a single UK centre looked at patients with the highest-risk presentations of RAS (flash pulmonary oedema, rapidly declining renal function, refractory hypertension) and compared the outcomes for both medical therapy and revascularisation in these patients with those for low-risk patients (those who had none of the above-mentioned features). In total, 467 patients were included, of whom 51 % had at least one high-risk feature. After a median 3.8-year follow-up period, patients presenting with flash pulmonary oedema had a significantly higher incidence of cardiovascular events and death; however, this risk was reduced if these patients underwent revascularisation (HR 0.43; 95 % CI 0.2 0.9). There were also non-significant trends towards improved outcomes in patients presenting with two or more high-risk features. The study authors acknowledged the limitations of their study using observational data and small patient numbers, but concluded that patients with flash pulmonary oedema and patients with more than one high-risk feature at presentation should be considered for revascularisation and further clinical trials in these high-risk groups were necessary [76].

## Conclusion

RAS secondary to FMD is probably commoner than previously thought. FMD should be actively looked for in young (particularly female) patients presenting with hypertension and should be considered in older patients with difficult-to-treat hypertension. Management with angioplasty is recommended in symptomatic patients, and outcomes are generally good, particularly in younger patients. RAS secondary to atherosclerosis is likely to be seen with increasing frequency owing to an aging population undergoing more imaging studies. Current best evidence supports aggressive medical management in these patients with good blood pressure control (including use of an RAAS antagonist) and a statin. Less evidence-based but probably reasonable additional treatment would be an antiplatelet agent and lifestyle advice. Revascularisation may be considered for a subset of patients who fail to stabilise with medical management or for patients who present with multiple high-risk features, particularly flash pulmonary oedema. Reliable, objective criteria for identifying patients who will benefit from revascularisation have yet to be established, and prospective trials in these subsets of patients are now needed.

## References

[1] Seddon M, Saw J (2011). Atherosclerotic renal artery stenosis: review of pathophysiology, clinical trial evidence, and management strategies. Can J Cardiol.

[2] Cooper CJ, Murphy TP, Cutlip DE, Jamerson K, Henrich W, Reid D (2014). Stenting and medical therapy for atherosclerotic renal-artery stenosis. N Engl J Med.

[3] Wheatley K, Ives N, Gray R, Kalra PA, Moss JG, Baigent C (2009). Revascularization versus medical therapy for renal-artery stenosis. N Engl J Med.

[4] Hansen KJ, Edwards MS, Craven TE, Cherr GS, Jackson SA, Appel RG (2002). Prevalence of renovascular disease in the elderly: a population-based study. J Vasc Surg.

[5] Jean WJ, Albitar I, Zwicke DL, Port SC, Schmidt DH, Bajwa TK (1994). High incidence of renal artery stenosis in patients with coronary artery disease. Catheter Cardiovasc Diagn.

[6] Missouris CG, Buckenham T, Cappuccio FP, Macgregor GA (1994). Renal artery stenosis: a common and important problem in patients with peripheral vascular disease. Am J Med.

[7] Persu A, Touze E, Mousseaux E, Barral X, Joffre F, Plouin PF (2011). Diagnosis and management of fibromuscular dysplasia: an expert consensus. Eur J Clin Investig.

[8] Begelman SM, Olin JW (2000). Fibromuscular dysplasia. Curr Opin Rheumatol.

[9] Kincaid OW, Davis GD, Hallerma FJ, Hunt JC (1968). Fibromuscular dysplasia of renal arteries. Arteriographic features classification and observations on natural history of disease. Am J Roentgenol Radium Ther Nucl Med.

[10] Trinquart L, Mounier-Vehier C, Sapoval M, Gagnon N, Plouin PF (2010). Efficacy of revascularization for renal artery stenosis caused by fibromuscular dysplasia: a systematic review and meta-analysis. Hypertension.

[11] Textor SC, Greco BA, Floege J, Johnson RJ, Feehally J (2010). Renovascular hypertension and ischaemic renal disease. Comprehensive clinical nephrology.

[12] Olin JW, Froehlich J, Gu XK, Bacharach JM, Eagle K, Gray BH (2012). The United States Registry for Fibromuscular Dysplasia: results in the first 447 patients. Circulation.

[13] Savard S, Azarine A, Jeunemaitre X, Azizi M, Plouin PF, Steichen O (2013). Association of smoking with phenotype at diagnosis and vascular interventions in patients with renal artery fibromuscular dysplasia. Hypertension.

[14] Blondin D, Lanzman R, Schellhammer F, Oels M, Grotemeyer D, Baldus SE (2010). Fibromuscular dysplasia in living renal donors: still a challenge to computed tomographic angiography. Eur J Radiol.

[15] Cragg AH, Smith TP, Thompson BH, Maroney TP, Stanson AW, Shaw GT (1989). Incidental fibromuscular dysplasia in potential renal donors - long-term clinical follow-up. Radiology.

[16] McKenzie GA, Oderich GS, Kawashima A, Misra S (2013). Renal artery fibromuscular dysplasia in 2,640 renal donor subjects: a CT angiography analysis. J Vasc Interv Radiol.

[17] De Bruyne B, Manoharan G, Pijls NHJ, Verhamme K, Madaric J, Bartunek J (2006). Assessment of renal artery stenosis severity by pressure gradient measurements. J Am Coll Cardiol.

[18] Textor SC (1984). Patho-physiology of renovascular hypertension. Urol Clin N Am.

[19] Renal Association (2012). UK Renal Registry. Fifteenth annual report.

[20] Mailloux LU, Napolitano B, Bellucci AG, Vernace M, Wilkes BM, Mossey RT (1994). Renal vascular disease causing end-stage renal disease, incidence, clinical correlates, and outcomes: a 20-vear clinical experience. Am J Kidney Dis.

[21] Messerli FH, Bangalore S, Makani H, Rimoldi SF, Allemann Y, White CJ (2011). Flash pulmonary oedema and bilateral renal artery stenosis: the Pickering syndrome. Eur Heart J.

[22] Hirsch AT, Haskal ZJ, Hertzer NR, Bakal CW, Creager MA, Halperin JL (2006). ACC/AHA 2005 practice guidelines for the management of patients with peripheral arterial disease (lower extremity, renal, mesenteric, and abdominal aortic): a collaborative report from the American Association for Vascular Surgery/Society for Vascular Surgery,* Society for Cardiovascular Angiography and Interventions, Society for Vascular Medicine and Biology, Society of Interventional Radiology, and the ACC/AHA Task Force on Practice Guidelines (Writing Committee to Develop Guidelines for the Management of Patients with Peripheral Arterial Disease). Circulation.

[23] Conlon PJ, Little MA, Pieper K, Mark DB (2001). Severity of renal vascular disease predicts mortality in patients undergoing coronary angiography. Kidney Int.

[24] Caps MT, Perissinotto C, Zierler RE, Polissar NL, Bergelin RO, Tullis MJ (1998). Prospective study of atherosclerotic disease progression in the renal artery. Circulation.

[25] Tollefson DFJ, Ernst CB (1991). Natural history of atherosclerotic renal artery stenosis associated with aortic disease. J Vasc Surg.

[26] Williams GJ, Macaskill P, Chan SF, Karplus TE, Yung W, Hodson EM (2007). Comparative accuracy of renal duplex sonographic parameters in the diagnosis of renal artery stenosis: paired and unpaired analysis. Am J Roentgenol.

[27] Olin JW, Piedmonte MR, Young JR, Deanna S, Grubb M, Childs MB (1995). The utility of duplex ultrasound scanning of the renal arteries for diagnosing significant renal artery stenosis. Ann Intern Med.

[28] Drieghe B, Madaric J, Sarno G, Manoharan G, Bartunek J, Heyndrickx GR (2008). Assessment of renal artery stenosis: side-by-side comparison of angiography and duplex ultrasound with pressure gradient measurements. Eur Heart J.

[29] Beregi JP, Louvegny S, Gautier C, Mounier-Vehier C, Moretti A, Desmoucelle F (1999). Fibromuscular dysplasia of the renal arteries: comparison of helical CT angiography and arteriography. Am J Roentgenol.

[30] Willoteaux S, Faivre-Pierret M, Moranne O, Lions C, Bruzzi J, Finot M (2006). Fibromuscular dysplasia of the main renal arteries: comparison of contrast-enhanced MR angiography with digital subtraction angiography. Radiology.

[31] Vasbinder GBC, Nelemans PJ, Kessels AGH, Kroon AA, Maki JH, Leiner T (2004). Accuracy of computed tomographic angiography and magnetic resonance angiography for diagnosing renal artery stenosis. Ann Intern Med.

[32] Rountas C, Vlychou M, Vassiou K, Liakopoulos V, Kapsalaki E, Koukoulis G (2007). Imaging modalities for renal artery stenosis in suspected renovascular hypertension: prospective intraindividual comparison of color Doppler US, CT angiography, GD-enhanced MR angiography, and digital substraction angiography. Ren Fail.

[33] Vasbinder GBC, Nelemans PJ, Kessels AGH, Kroon AA, de Leeuw PW, van Engelshoven JMA (2001). Diagnostic tests for renal artery stenosis in patients suspected of having renovascular hypertension: a meta-analysis. Ann Intern Med.

[34] Gandy SJ, Sudarshan TAP, Sheppard DG, Allan LC, McLeay TB, Houston JG (2003). Dynamic MRI contrast enhancement of renal cortex: a functional assessment of renovascular disease in patients with renal artery stenosis. J Magn Reson Imaging.

[35] Gillis KA, McComb C, Foster JE, Taylor AH, Patel RK, Morris ST (2014). Inter-study reproducibility of arterial spin labelling magnetic resonance imaging for measurement of renal perfusion in healthy volunteers at 3 Tesla. BMC Nephrol.

[36] Tan H, Koktzoglou I, Prasad PV (2014). Renal perfusion imaging with two-dimensional navigator gated arterial spin labeling. Magn Reson Med.

[37] Gandy SJ, Armoogum K, Nicholas RS, McLeay TB, Houston JG (2007). A clinical MRI investigation of the relationship between kidney volume measurements and renal function in patients with renovascular disease. Br J Radiol.

[38] Lim SW, Chrysochou C, Buckley DL, Kalra PA, Sourbron SP (2013). Prediction and assessment of responses to renal artery revascularization with dynamic contrast-enhanced magnetic resonance imaging: a pilot study. Am J Physiol Ren Physiol.

[39] Chrysochou C, Mendichovszky IA, Buckley DL, Cheung CM, Jackson A, Kalra PA (2012). BOLD imaging: a potential predictive biomarker of renal functional outcome following revascularization in atheromatous renovascular disease. Nephrol Dial Transplant.

[40] Zhang JL, Morrell GR, Lee VS (2013). Blood oxygen level-dependent MR in renal disease: moving toward clinical utility. Radiology.

[41] Ramsay LE, Waller PC (1990). Blood-pressure response to percutaneous transluminal angioplasty for renovascular hypertension - an overview of published series. Br Med J.

[42] Smit JV, Wierema TKA, Kroon AA, de Leeuw PW (2013). Blood pressure and renal function before and after percutaneous transluminal renal angioplasty in fibromuscular dysplasia: a cohort study. J Hypertens.

[43] Slovut DP, Olin JW (2004). Current concepts - fibromuscular dysplasia. N Engl J Med.

[44] van de Ven PJG, Beutler JJ, Kaatee R, Beek FJA, Mali W, Koomans HA (1998). Angiotensin converting enzyme inhibitor-induced renal dysfunction in atherosclerotic renovascular disease. Kidney Int.

[45] Chrysochou C, Foley RN, Young JF, Khavandi K, Cheung CM, Kalra PA (2012). Dispelling the myth: the use of renin-angiotensin blockade in atheromatous renovascular disease. Nephrol Dial Transplant.

[46] Losito A, Errico R, Santirosi P, Lupattelli T, Scalera GB, Lupattelli L (2005). Long-term follow-up of atherosclerotic renovascular disease. Beneficial effect of ACE inhibition. Nephrol Dial Transplant.

[47] Hackam DG, Duong-Hua ML, Mamdani M, Li P, Tobe SW, Spence JD (2008). Coronary artery disease - angiotensin inhibition in renovascular disease: a population-based cohort study. Am Heart J.

[48] Chade AR, Zhu XY, Grande JP, Krier JD, Lerman A, Lerman LO (2008). Simvastatin abates development of renal fibrosis in experimental renovascular disease. J Hypertens.

[49] Bates MC, Campbell JE, Stone PA, Jaff MR, Broce M, Lavigne PS (2007). Factors affecting long-term survival following renal artery stenting. Catheter Cardiovasc Interv.

[50] Silva VS, Martin LC, Franco RJS, Carvalho FC, Bregagnollo EA, Castro JH (2008). Pleiotropic effects of statins may improve outcomes in atherosclerotic renovascular disease. Am J Hypertens.

[51] Cheung CM, Patel A, Shaheen N, Cain S, Eddington H, Hegarty J (2007). The effects of statins on the progression of atherosclerotic renovascular disease. Nephron Clin Pract.

[52] Jonason T, Bergstrom R (1987). Cessation of smoking in patients with intermittent claudication - effects on the risk of peripheral vascular complications, myocardial infarction and mortality. Acta Med Scand.

[53] Antiplatelet Trialists’ Collaboration. Collaborative overview of randomized trials of antiplatelet therapy—I: Prevention of death, myocardial-infarction and stroke by prolonged antiplatelet therapy in various categories of patients. Br Med J. 1994;308(6943):1540. PMC2539220 8298418

[54] Antiplatelet Trialists’ Collaboration (1994). Collaborative overview of randomized trials of antiplatelet therapy—II: maintenance of vascular graft or arterial patency by antiplatelet therapy. Br Med J.

[55] Girolami B, Bernardi E, Prins MH, ten Cate JW, Prandoni P, Hettiarachchi R (1999). Antithrombotic drugs in the primary medical management of intermittent claudication: a meta-analysis. Thromb Haemost.

[56] Anand S, Yusuf S, Xie C, Pogue J, Eikelboom J, Budaj A (2007). Oral anticoagulant and antiplatelet therapy and peripheral arterial disease. N Engl J Med.

[57] Weibull H, Bergqvist D, Bergentz SE, Jonsson K, Hulthen L, Manhem P (1993). Percutaneous transluminal renal angioplasty versus surgical reconstruction of atherosclerotic renal artery stenosis: a prospective randomized study. J Vasc Surg.

[58] van de Ven PJG, Kaatee R, Beutler JJ, Beek FJA, Woittiez AJJ, Buskens E (1999). Arterial stenting and balloon angioplasty in ostial atherosclerotic renovascular disease: a randomised trial. Lancet.

[59] Burket MW, Cooper CJ, Kennedy DJ, Brewster PS, Ansel GM, Moore JA (2000). Renal artery angioplasty and stent placement: predictors of a favorable outcome. Am Heart J.

[60] Rocha-Singh K, Jaff MR, Rosenfield K (2005). Evaluation of the safety and effectiveness of renal artery stenting after unsuccessful balloon angioplasty - the ASPIRE-2 study. J Am Coll Cardiol.

[61] Murphy TP, Soares G, Kim M (2004). Increase in utilization of percutaneous renal artery interventions by Medicare beneficiaries, 1996-2000. Am J Roentgenol.

[62] Liang P, Hurks R, Bensley RP, Hamdan A, Wyers M, Chaikof E (2013). The rise and fall of renal artery angioplasty and stenting in the United States, 1988-2009. J Vasc Surg.

[63] Dworkin LD, Murphy T (2010). Is there any reason to stent atherosclerotic renal artery stenosis?. Am J Kidney Dis.

[64] Webster J, Marshall F, Abdalla M, Dominiczak A, Edwards R, Isles CG (1998). Randomised comparison of percutaneous angioplasty vs continued medical therapy for hypertensive patients with atheromatous renal artery stenosis. J Hum Hypertens.

[65] van Jaarsveld BC, Krijnen P, Pieterman H, Derkx FHM, Deinum J, Postma CT (2000). The effect of balloon angioplasty on hypertension in atherosclerotic renal-artery stenosis. N Engl J Med.

[66] Bax L, Woittiez A-JJ, Kouwenberg HJ, Mali WPTM, Buskens E, Beek FJA (2009). Stent placement in patients with atherosclerotic renal artery stenosis and impaired renal function: a randomized trial. Ann Intern Med.

[67] Balk E, Raman G, Chung M, Ip S, Tatsioni A, Alonso A (2006). Comparative effectiveness of management strategies for renal artery stenosis. Comparative effectiveness review no .5.

[68] Cooper CJ, Murphy TP, Matsumoto A, Steffes M, Cohen DJ, Jaff M (2006). Stent revascularization for the prevention of cardiovascular and renal events among patients with renal artery stenosis and systolic hypertension: rationale and design of the CORAL trial. Am Heart J.

[69] Messina LM, Zelenock GB, Yao KA, Stanley JC (1992). Renal revascularization for recurrent pulmonary edema in patients with poorly controlled hypertension and renal insufficiency: a distinct subgroup of patients with arteriosclerotic renal artery occlusive disease. J Vasc Surg.

[70] Gray BH, Olin JW, Childs MB, Sullivan TM, Bacharach JM (2002). Clinical benefit of renal artery angioplasty with stenting for the control of recurrent and refractory congestive heart failure. Vasc Med.

[71] Bloch MJ, Trost DW, Pickering TG, Sos TA, August P (1999). Prevention of recurrent pulmonary edema in patients with bilateral renovascular disease through renal artery stent placement. Am J Hypertens.

[72] van den Berg DTNA, Deinum J, Postma CT, van der Wilt GJ, Riksen NP (2012). The efficacy of renal angioplasty in patients with renal artery stenosis and flash oedema or congestive heart failure: a systematic review. Eur J Heart Fail.

[73] Mukherjee D (2009). Renal artery revascularization. Is there a rationale to perform?. JACC Cardiovasc Interv.

[74] Textor SC, Lerman LO (2014). Reality and renovascular disease: when does renal artery stenosis warrant revascularization?. Am J Kidney Dis.

[75] Lao D, Parasher PS, Cho KC, Yeghiazarians Y (2011). Atherosclerotic renal artery stenosis—diagnosis and treatment. Mayo Clin Proc.

[76] Ritchie J, Green D, Chrysochou C, Chalmers N, Foley RN, Kalra PA (2014). High-risk clinical presentations in atherosclerotic renovascular disease: prognosis and response to renal artery revascularization. Am J Kidney Dis.

